# Supplementary material for: The causal relationship between the human gut microbiota and pyogenic arthritis: a Mendelian randomization study
Source: Front Cell Infect Microbiol. 2024 Nov 26;14:1452480. doi: 10.3389/fcimb.2024.1452480 (PMC11629706; doi:10.3389/fcimb.2024.1452480)
Supplement: Supplementary file 1 [file DataSheet1.zip › mendelian test/ebi-a-GCST90027827.csv_leave_one_out.pdf]

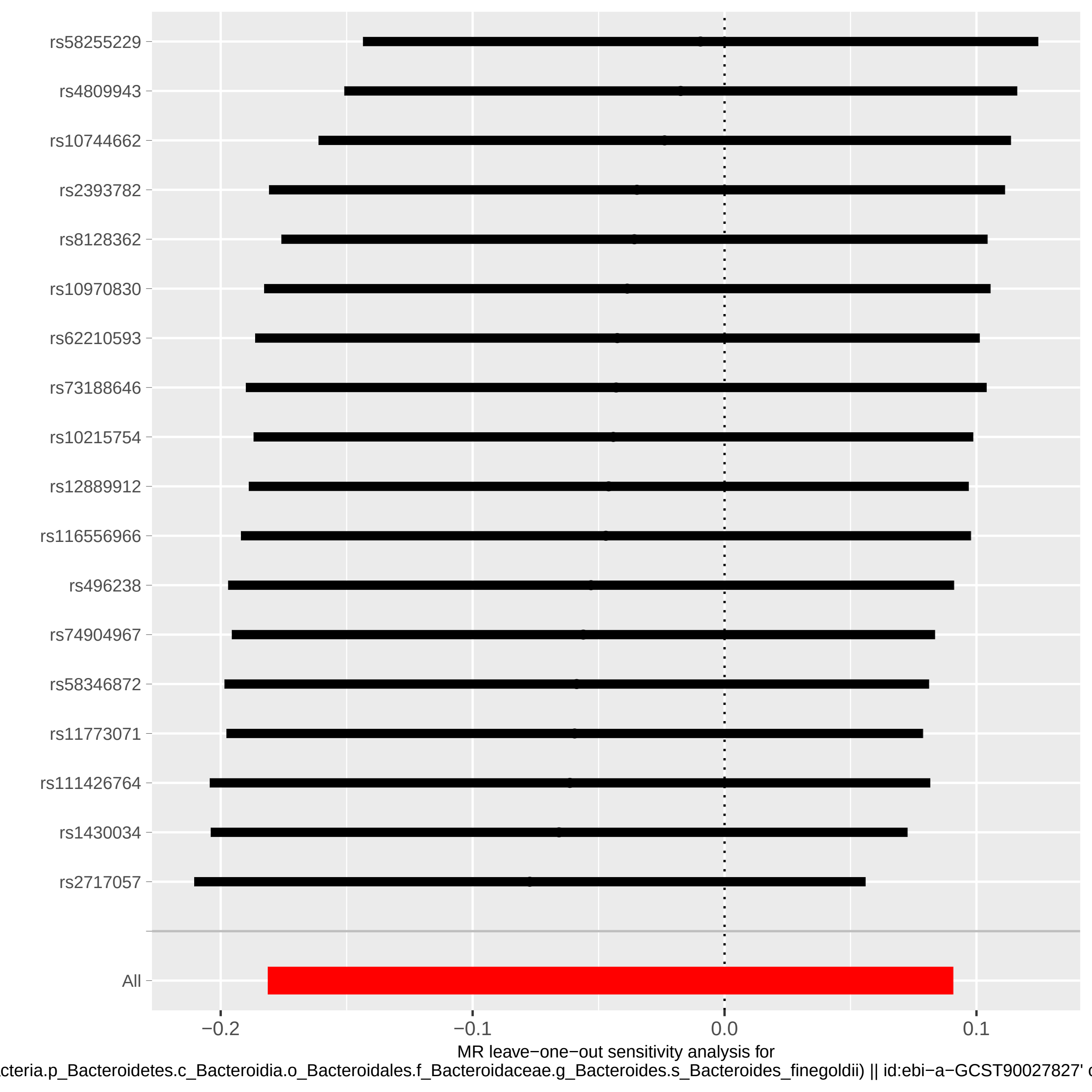
rs58255229
rs4809943
rs10744662
rs2393782
rs8128362
rs10970830
rs62210593
rs73188646
rs10215754
rs12889912
rs116556966
rs496238
rs74904967
rs58346872
rs11773071
rs111426764
rs1430034
rs2717057
All
−0.2
−0.1
0.0
0.1
MR leave−one−out sensitivity analysis for
cteria.p_Bacteroidetes.c_Bacteroidia.o_Bacteroidales.f_Bacteroidaceae.g_Bacteroides.s_Bacteroides_finegoldii) || id:ebi−a−GCST90027827' o
